# CD6 expression has no effect on atherosclerosis in apolipoprotein E-deficient mice

**DOI:** 10.1186/s13104-018-3327-4

**Published:** 2018-04-03

**Authors:** Juying Han, Gospel Enyindah-Asonye, Feng Lin, Jonathan D. Smith

**Affiliations:** 1 Department of Cellular & Molecular Medicine, Lerner Research Institute, Cleveland Clinic, 9500 Euclid Avenue, Cleveland, OH USA; 2 Department of Immunology, Lerner Research Institute, Cleveland Clinic, 9500 Euclid Avenue, Cleveland, OH USA

**Keywords:** B1a cells, Natural IgM, Atherosclerosis, CD6

## Abstract

**Objective:**

To determine if deficiency of CD6, a cell surface protein on lymphocytes that alters natural antibody production, increases atherosclerosis in ApoE-deficient mice fed a chow or a western-type diet.

**Results:**

We compared cholesterol levels, IgM, B1a cells, and aortic root lesion areas in ApoE-deficient vs. CD6/ApoE double deficient mice. Feeding the high-fat western type diet increased all parameters, except for B1a cell numbers decreased. Sex also had an effect on many parameters with males having increased body weights, higher high density lipoprotein cholesterol, higher B1a cells, but smaller atherosclerotic lesions if chow fed mice; however, this sex effect on atherosclerosis was absent in mice fed the western-type diet. CD6 deficiency had no effect on atherosclerosis in both male and female mice on either diet. Thus, loss of CD6 on lymphocytes did not lead to expected reductions in B1a cells and protective IgM levels, and in turn did not alter atherosclerosis in mice.

## Introduction

Atherosclerosis is a chronic inflammatory disease of the artery wall as well as the leading cause of heart attacks and strokes, and B cell subsets can alter atherosclerosis pathogenesis [1–3]. Previous studies have uncovered a protective role for the B1a cells in atherosclerosis [4–6]. Therefore, a better understanding of molecules restricted to B1a cells may lead to the development of novel therapeutics against atherosclerosis.

B cells can be divided into B1 and B2 subtypes based on their developmental origin, function and phenotypic markers [7, 8]. B1 cells can be further divided into B1a, CD5 positive, and B1b, CD5 negative, subsets [9, 10]. B1a cells spontaneously secrete natural IgM which is atheroprotective in splenectomized apolipoprotein E (apoE)-deficient mice [11]. These mice are susceptible to atherosclerosis in association with reduced B1a cells/natural IgM titers, but display amelioration of atherosclerosis following reconstitution with B1a or with purified natural IgM from naïve wildtype mice, but not with B2 cells [5, 12].

CD6 is primary expressed on T cells and a subset of B cells in humans [13–15]. In mouse B1 cells, CD6 is only expressed in non-bone marrow and peritoneal B1a subsets [16]. CD6 is important for B1a cell homeostasis and natural IgM levels in mice. CD6 deficiency resulted in reduced serum titers of natural IgM and reduced B1a cells in select-tissue compartments [16]. CD6-deficient mice are also protected from natural IgM-mediated intestinal ischemia reperfusion-induced injury [16].

We generated the double knockout (DKO) mouse model of CD6 deficiency (CD6−/−) and apolipoprotein E deficiency (apoE−/−) on the DBA atherosclerosis-sensitive genetic background [17] in order to determine if CD6 expression is atheroprotective due to increased production of natural IgM.

## Main text

### Methods

#### Generation of CD6 and ApoE double knockout mice

ApoE−/− mice [18] were previously backcrossed onto the DBA/2 background for > 20 generations [17]. CD6−/− mice were originally made at Bristol-Myers Squibb and backcrossed onto the DBA/1 background for > 12 generations [19]. These strains were intercrossed to generate F1 mice hemizygous for both ApoE and CD6, which were bred back to DBA/2 ApoE−/− mice to select ApoE−/− CD6+/− progeny. These ApoE−/− CD6+/− mice were brother–sister mated to generate CD6+/+, CD6+/−, and CD6−/− mice on the ApoE−/− background. We used the CD6+/+ and CD6−/− mice in these studies.

#### Atherosclerosis assay

ApoE−/− CD6+/+ (KO) and ApoE−/− CD6−/− (double knockout, DKO) mice were fed a chow diet (Teklad 2018) or a western-type diet (WTD, Teklad 88137, 21% milk fat/0.15% cholesterol (wt:wt), starting at 8 weeks of age). Mice were sacrificed by CO_2_ inhalation at 16 weeks of age. Whole blood was collected from the retro-orbital plexus to obtain plasma. Quantitative assessment of atherosclerosis in the aortic root was performed as previously described [20]. Lesion areas were quantified as the mean value in six sections at 80 μm intervals using Image Pro software (Media Cybernetics). We analyzed 14 KO and 6 DKO male mice and 16 KO and 10 DKO female mice on chow diet; and, 8 KO and 6 DKO male mice and 5 KO and 10 DKO female mice on WTD. Atherosclerosis assays of three female KO on chow and one DKO on WTD diet were not able to be quantified due to improper sectioning through the aortic root, yielding 13 and 9 samples respectively.

#### Determination of total cholesterol and HDL-cholesterol

30 μl of plasma was mixed with 30 μl of KBr (density of 1.12 g/ml), placed into 0.2 ml tubes and centrifuged at 70,000 rpm for 16 h in an S100-AT3 rotor (Thermo Scientific). The bottom 30 μl layer with a density > 1.063 was used to determine HDL-cholesterol, and whole plasma was used for total cholesterol levels, both run with technical duplicates using the Cholesterol Liquicolor kit (StanBio Laboratory, catalog#1010-225). Missing values were due to insufficient plasma recovery or poor ultracentrifugation.

#### B cell population determined by flow cytometry

Mouse fresh spleens were removed after PBS perfusion. Single-cell suspensions were prepared by slicing the excised spleen into small pieces and passage through a strainer. Following centrifugation of the cell suspension, the cell pellet was incubated with red blood cell lysing solution for 3 min. The splenocytes were washed with PBS and incubated with the following antibodies: anti-CD19 (catalog#115532); anti-CD43 (catalog#143206); anti-CD5 (catalog#100626); and, anti-CD6 clone 34 (prepared in the Lin laboratory). All antibodies except for anti-CD6 were acquired from BioLegend and diluted 1/100. Cells were stained in FACS buffer, and expression of cell surface markers was acquired on the FACSCalibur flow cytometer. Flow cytometer data were analyzed using Flowjo software. Missing values were due to insufficient blood recovery or no access to the flow cytometer when the fresh stained cells were prepared.

#### Plasma IgM and phosphorylcholine specific IgM (PC-IgM) assays

Total IgM were detected by coating 96-well plates with 0.34 μg/ml rabbit anti-mouse IgM+IgG (H+L) (Jackson ImmunoResearch, catalog#315-005-048). Plasma samples (diluted 1/1000) were added to the plates. Bound antibodies were detected using goat anti-mouse IgM HRP (Southern Biotech, catalog#1021-05). For the detection of PC-IgM, plates were coated with 10 μg/ml PC-BSA (Biosearch Technologies, catalog#PC-1011-10). Samples (diluted 1/1000) were added and bound PC-IgM antibodies were detected using goat anti-mouse IgM-HRP (Southern Biotech, catalog#1021-05). The ELISA was developed using TMB substrate (Pierce, catalog#34021). H_2_SO_4_ was added to stop the reaction and absorbance at 450 nm was measured. Missing values were due to insufficient plasma recovery.

#### Statistical analyses

All phenotypic data was analyzed using a number code that did not specify genotype or sex, thus data acquisition was blinded. All data were normally distributed passing the KS and/or D’Agostino and Pearson normality tests, and parametric statistics were used. Results are expressed as the mean ± SD. T-tests were used for comparisons of KO vs DKO phenotypes within one diet and sex. Two way ANOVAs with Bonferroni correction for multiple comparisons were performed to analyze diet and sex effects simultaneously with genotype effects. P values < 0.05 were considered significant. Statistical analyses were performed using Prism v5.04 software (GraphPad).

### Results

#### Characterization of DKO mice

We crossed CD6−/− mice on the DBA/1 background to ApoE−/− on the closely related DBA/2 background, and two groups of mice were studied: KO and DKO. As sex is an important biological variable, we analyzed all phenotypes separately by sex.

The genotype and diet effects in male mice are shown in Table 1. On the chow diet, there were no significant differences between the KO and DKO for any of the measured phenotypes. On the WTD, total IgM levels were significantly higher in the DKO vs. KO mice, but no other measures were significantly different. We looked for diet and genotype effects using two-way ANOVA. WTD had significant effects on all of the phenotypes, increasing body weight, total and HDL cholesterol, IgM, PC-IgM, and lesion area, while decreasing B1a cells. The genotype effect was limited to PC-IgM, which was higher in the DKO mice. We found no significant interaction between the diet and genotype effects in the male mice.

**Table 1:** CD6 genotype and diet effects in male mice

Phenotype	Chow diet			Western type diet			Diet effect	Genotype	Interaction
	K0	DK0	T-test	K0	DK0	T-test	P value	P value	P value
Male mice									
Body weight (g)	28.3 ± 3.2 (14)	28 ± 1.5 (6)	N.S	35.5 ± 4.0 (8)	31.8 ± 3.4 (6)	N.S	*< 0.0001*	N.S	N.S
Cholesterol (mg/dl)	1251 ± 161.1 (10)	1179 ± 143.1 (5)	N.S	2518 ± 257.5 (8)	2876 ± 507.7 (6)	N.S	*< 0.0001*	N.S	N.S
HDL-C (mg/dl)	86 ± 26.3 (8)	79 ± 28.2 (5)	N.S	146 ± 88.4 (8)	130.2 ± 63.7 (6)	N.S	*0.026*	N.S	N.S
IgM (μg/ml)	55.6 ± 33.5 (14)	54.3 ± 27.1 (6)	N.S	70.2 ± 29.8 (8)	102.3 ± 16.3 (6)	*0.036*	*0.006*	N.S	N.S
Pc-IgM (OD read)	0.15 ± 0.1 (14)	0.21 ± 0.11 (5)	N.S	0.51 ± 0.22 (8)	0.7 ± 0.21 (6)	N.S	*< 0.0001*	*0.042*	N.S
B1a cells (% of total cells)	4.17 ± 1.43 (10)	3.96 ± 0.8 (5)	N.S	1.8 ± 1.22 (5)	2.93 ± 1.36 (6)	N.S	*0.0026*	N.S	N.S
Lesion area (μm^2^)	114,960 ± 52,300 (14)	95,595 ± 43,576 (6)	N.S	507,311 ± 209,049 (8)	674,382 ± 292,443 (6)	N.S	*< 0.0001*	N.S	N.S

The genotype and diet effects in female mice are shown in Table 2. On the chow diet, there were no significant differences between the KO and DKO for any of the measured phenotypes. On the WTD, B1a cells was significantly decreased in the DKO vs. KO mice, but no other measures were significantly different. In the two-way ANOVA analysis, WTD had significant effects on increasing body weight, total cholesterol, PC-IgM, and lesion area, while decreasing HDL-C and B1a cells. The genotype effect was limited to B1a cell number, which were lower in the DKO mice. We found a significant interaction between the diet and genotype effects for both PC-IgM and lesion area in the female mice.

**Table 2:** CD6 genotype and diet effects in female mice

Phenotype	Chow diet			Western type diet			Diet effect	Genotype	Interaction
	K0	DK0	T-test	K0	DK0	T-test	P value	P value	P value
Female mice									
Body weight (g)	23.9 ± 2.5 (16)	23.4 ± 2.9 (10)	N.S	28.4 ± 5.2 (5)	25.9 ± 3.5 (10)	N.S	*0.003*	N.S	N.S
Cholesterol (mg/dl)	1064 ± 159.8 (13)	1127 ± 240.9 (7)	N.S	2338 ± 332.4 (5)	2040 ± 473.7 (10)	N.S	*< 0.0001*	N.S	N.S
HDL-C (mg/dl)	56.6 ± 14.4 (10)	53.8 ± 16.2 (7)	N.S	40.5 ± 27.6 (3)	44.2 ± 24.0 (7)	N.S	N.S	N.S	N.S
IgM (μg/ml)	56.8 ± 47.4 (14)	65.4 ± 34.1 (10)	N.S	74.63 ± 21 (5)	63.8 ± 24.4 (9)	N.S	N.S	N.S	N.S
Pc-IgM (OD read)	0.31 ± 0.2 (14)	0.18 ± 0.13 (10)	N.S	0.54 ± 0.25 (5)	0.85 ± 0.46 (10)	N.S	*< 0.0001*	N.S	*0.032*
B1a cells (% of total cells)	3.16 ± 0.58 (7)	2.49 ± 1.23 (6)	N.S	1.97 ± 0.54 (4)	1.29 ± 0.45 (7)	*0.049*	*0.0013*	*0.048*	N.S
Lesion area (μm^2^)	129,119 ± 59,493 (13)	185,825 ± 108,327 (10)	N.S	596,416 ± 233,19 9(5)	466,142 ± 165,955 (9)	N.S	*< 0.0001*	N.S	*0.026*

Sex effects were examined combining both genotypes in mice fed the chow and WTD (Table 3). On chow diet, males had significantly higher body weights, HDL-C, and B1a cells, but lower lesion areas, the latter finding is common for aortic root lesion area in apoE−/− mice [21]. On the WTD, males had significantly higher body weights, total cholesterol, HDL-C, and a non-significant trend towards higher B1a cell number and lesion area.

**Table 3:** Sex effects in mice fed chow and western-type diets

Phenotype	Sex effect chow	Sex effect WTD
	P value	P value
Body weight (g)	< *0.0001*	*0.0002*
Cholesterol (mg/dl)	0.07	*< 0.0001*
HDL-C (mg/dl)	*0.0018*	*0.0013*
IgM (μg/ml)	0.61	0.08
Pc-IgM (OD read)	0.19	0.49
B1a cells (% of total cells)	*0.0006*	0.1
Lesion area (μm^2^)	*0.027*	0.49

### Discussion

We generated ApoE/CD6 DKO mice to address the role of CD6 in cardiovascular disease given the recent focus of CD6 as a therapeutic target for the treatment of several autoimmune diseases [16, 19, 22–25]. We hypothesized that the DKO mice would have larger lesions due to decreased B1a cells and natural IgM. In this study we report that DKO vs. KO did not significantly alter aortic root atherosclerosis in either male or female mice fed the chow or WTD. There were no significant differences between the two genotypes for most of the measured phenotypes; however, feeding WTD had significant effects on almost all of the phenotypes, and there were also many sex effects. Thus, the lack of altered atherosclerosis in the DKO mice indicates that CD6 therapeutic potential may not extend to cardiovascular-related disease.

We previously reported that splenic B1a cells and total IgM were decreased in chow diet-fed CD6−/− mice on the DBA/1 background [16]. After breeding to hyperlipidemic ApoE−/− mice on the DBA/2 background, B1a cells were not significantly changed in male mice on either diet, or in female mice on the chow diet, but were significantly decreased in female mice fed the WTD. Baseline splenic B1a cell levels were higher in ApoE−/− mice (~ 2–4%) compared to our prior study in wild type DBA/1 mice (1.4%), and perhaps the loss of CD6 was not as effective in reducing B1a cells in the context of elevated B1a cells in hyperlipidemic mice. The decrease in B1a cells in CD6−/− mice on the DBA/1 background was associated with moderate decreases in total IgM and PC-IgM [16]; however, with little effect on B1a cells in the current study, we found no effects on IgM levels, except for a moderate increase in total IgM in the male DKO mice fed the WTD. Previously, adoptive transfer of B1a cells into splenectomized apoE−/− mice fed the WTD increased B1a cells, IgM and slightly decreased lesion area [4]. However, CD6-deficiency did not significantly alter these parameters, having no effect on the protective PC-IgM levels; and, we did not observe an effect on atherosclerosis in the DKO mice. All IgM is not atheroprotective, only a subset of natural IgM has this property. Natural antibody PC-IgM has been reported to be protective in human cardiovascular disease and atherosclerosis [26, 27]. Further studies will be required to unravel the reasons for B1a-cell related phenotypic differences between KO and DKO mice.

Feeding the WTD led to significant effects in KO and DKO male mice, increasing body weight, total cholesterol, HDL-C, IgM, PC-IgM, and lesion area, while decreasing B1a cells. Similar effects of the WTD were seen in the female mice, although HDL-C and IgM levels trended up but were not significant. Thus, this challenge with the WTD uncovered an unexpected inverse association between endogenous B1a cells in the spleen and IgM/Pc-IgM levels, regardless of CD6 genotype. These findings were novel given that the effect of diet on CD6 mediated function has not been previously reported. However, B1a cell-mediated function has been reported to be influenced by environmental factors [28–31], and diet may contribute to the inverse association between B1a cells and IgM levels.

Sex effects on lipoprotein metabolism and atherosclerosis in mice are commonly observed, but in the opposite direction of what is seen in humans [21]. Aortic root lesion areas were greater in female than male mice that were fed the chow diet. However, this sex-effect was not found in the larger lesions observed after feeding the WTD, as the high total cholesterol values in WTD-fed mice may override this effect.

### Limitations

This study used CD6 and ApoE deficient mice that were on slightly different genetic backgrounds (DBA/1 and DBA/2, respectively), which could alter our results. A potential way to address this limitation in future studies could be to use anti-CD6 neutralizing antibody treatment in an inbred mouse model of atherosclerosis.

## Abbreviations

ApoE: apolipoprotein E
KO: ApoE knockout
DKO: ApoE/CD6 double knockout
WTD: western-type diet
PC-IgM: phosphorylcholine specific IgM
HDL-C: HDL cholesterol
